# Growth of large crystals of Janus phase RhSeCl using self-selecting vapour growth

**DOI:** 10.1039/d5ce01170a

**Published:** 2026-01-30

**Authors:** Anastasiia Lukovkina, Maria A. Herz, Xiaohanwen Lin, Volodymyr Multian, Alberto F. Morpurgo, Enrico Giannini, Fabian O. von Rohr

**Affiliations:** a Department of Quantum Matter Physics, University of Geneva 24 Quai Ernest-Ansermet CH-1211 Geneva Switzerland fabian.vonrohr@unige.ch +41 22 379 64 78

## Abstract

In recent years, interest in 2D Janus materials has grown exponentially, particularly with regard to their applications in spintronics and optoelectronic devices. The defining feature of Janus materials is the ordered arrangement of different layer terminations – creating chemically distinct surfaces and an inherent out-of-plane polarity. Among the few known Janus materials, RhSeCl is particularly intriguing as a rare example of an intrinsic Janus compound. Owing to its exceptional chemical stability, RhSeCl offers a promising platform for exploring the physics related to the Janus-structure. However, synthesising large, high-quality crystals of this compound remains a significant challenge. Here, we report a novel synthetic pathway for growing crystals up to 6 mm in lateral size *via* a two-step self-selecting vapour growth reaction. We further present a comprehensive comparison of newly developed synthesis routes with all previously reported methods for RhSeCl. During these investigations, we identified a previously unreported impurity that forms in specific growth pathways and demonstrate how it can be avoided to obtain phase-pure few- and monolayer flakes. We showcase the reproducibility of the process to obtain high-quality, large single-crystals and flakes.

## Abstract 2

Large Janus RhSeCl crystals grown *via* a two-step self-selecting vapour growth approach starting from SeCl_4_ and their exfoliation down to a monolayer for device fabrication.

## Graphical abstract

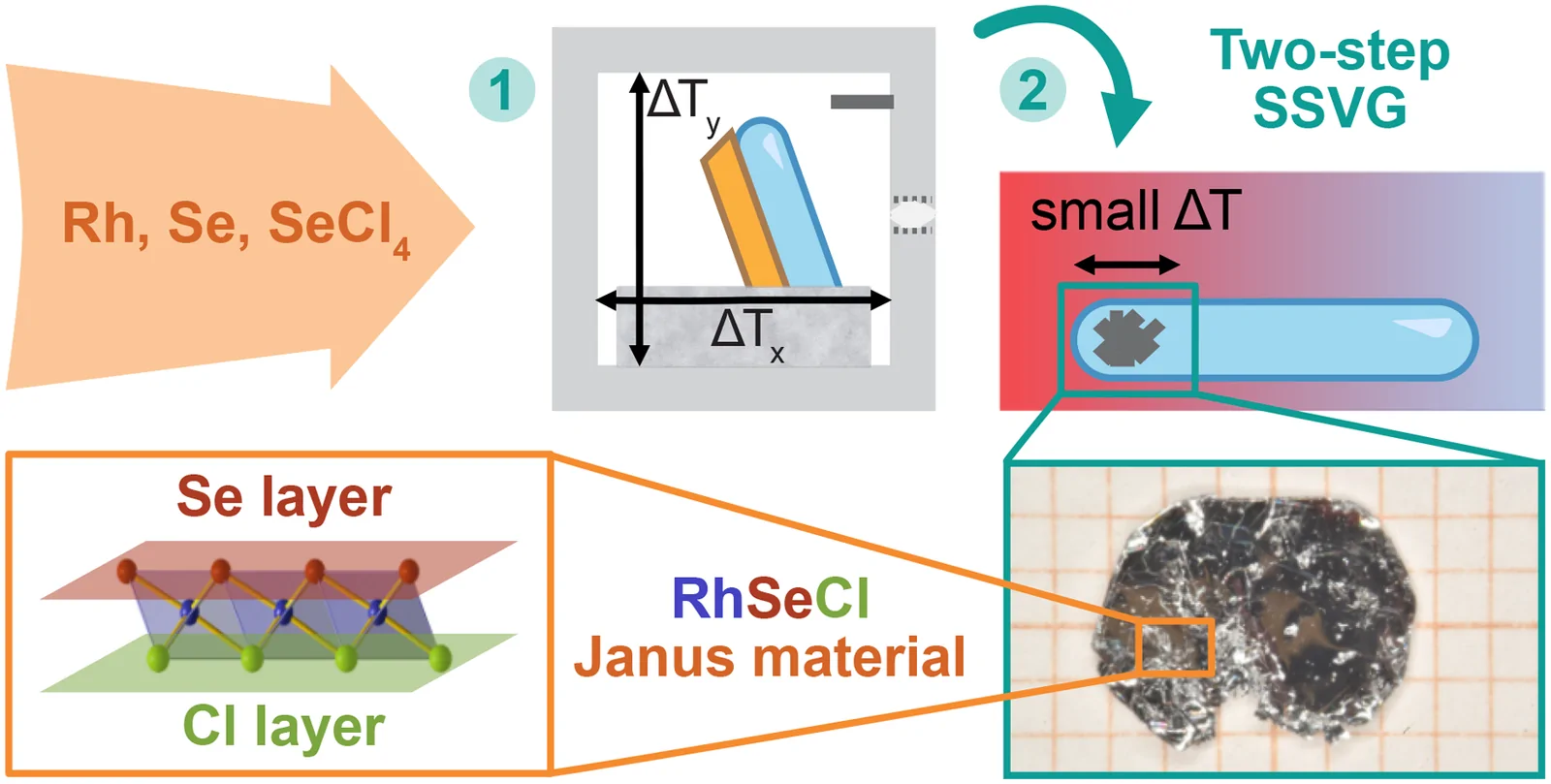

## 1 Introduction

Mixed-anion materials, in which two or more different anions coexist within one crystal structure at separate crystallographic sites, have emerged as a versatile platform for designing functional materials with tunable physical properties.^1,2^ Their chemical flexibility enables fine control over electronic structure, bonding, and symmetry – leading to phenomena such as ferroelectricity, Rashba splitting, and nonlinear optical effects.^3–5^

Mixed-anion van der Waals (vdW) materials have recently attracted increasing interest, as this particular type of chemistry enables controlled structural symmetry breaking.^6^ Representative examples include the magnetic semiconductor CrSBr,^7–9^ which displays quasi-one-dimensional transport properties, and CrPS_4_, a layered thiophosphate antiferromagnet with weak and tunable magnetic anisotropy, as well as a pronounced structural and optical anisotropy.^10,11^ Of particular interest are intrinsic Janus materials, a distinct type of mixed-anion compound in which different anions occupy opposite sides of each layer, breaking the local inversion symmetry of the central atoms.^12,13^ This separation due to the different determination of the layers leads to an internal electric field, as well as Rashba splitting, and piezoelectricity.^14^

The properties of two-dimensional Janus materials were first predicted theoretically and then studied experimentally using a bottom-up approach based on layer engineering. This was realised by a multitude of ways: through asymmetric modification of graphene with halogen atoms or organic molecules with various functional groups;^15–17^ by replacing one layer of S or Se atoms in a monolayer of MoSe_2_ or MoS_2_, respectively, to obtain MoSeS;^13,18,19^ and through layer-by-layer deposition using epitaxial techniques.^20^

The top-down approach, in which bulk crystals can be exfoliated down to a monolayer, is a simple and inexpensive method, but it can only be implemented if large, high-quality crystals of intrinsic Janus materials are grown. Only a few Janus compounds have been discovered so far that crystallise directly with separated ordered anionic layers. Among them are a family of bismuth chalcohalides^21–23^ and breathing Kagome Nb_3_ChI_7_ (with Ch = Se, Te).^24,25^ In addition to these materials, a further compound, RhSeCl, has recently been found to crystallise in a van der Waals layered structure, in the space group *P*6_3_*mc*, with separated layer determinations of chlorine and selenium anions, *i.e.*, in a Janus structure. This phase was initially discovered in 2018 as an analogue to RhTeCl,^26^ and was later identified as a noncentrosymmetric 2D Janus material in 2023.^27^

The growth of large RhSeCl crystals has proven to be difficult, and the reported syntheses produce only polycrystalline powders or small intergrown crystals. The initial successful approach in 2018 departed from employing AlCl_3_ as a chlorine source as used in RhTeCl, and instead was completed as a one-step solid-state reaction employing merely Se and RhCl_3_, see eqn (1).^26^ To avoid the formation of SeCl_4_ and Se_2_Cl_2_, Nowak *et al.* adapted this to a chemical-vapour-transport (CVT) synthesis and introduced elemental Rh into the reaction, see eqn (2). In their CVT, they employed a sink temperature *T*_1_ = 900 °C and source temperature *T*_2_ = 1000 °C, as well as a small excess of RhCl_3_.^27^ In addition to this conventional CVT approach with a static temperature profile, they performed crystal growth from the gas phase with 27 temperature oscillation cycles.^28^ In early 2025, Liu *et al.* adapted the CVT approach by adjusting the RhCl_3_ excess to 10% to enhance the transport rate of the crystals, and changing the sink temperature to *T*_1_ = 930 °C with maintaining *T*_2_ = 1000 °C as they found that a smaller Δ*T* of 70 °C reduced the thermal stress and the formation of defects in the crystals during growth.^29^ All the methods resulted in up to 1 mm laterally-sized crystals.^27–29^

1 2RhCl_3_ + 3Se → 2RhSeCl + SeCl_4_

2 2Rh + RhCl_3_ + 3Se → 3RhSeCl

Here, we present a systematic study of the synthesis and crystal growth of the Janus compound RhSeCl. Understanding and exploiting the properties of this intrinsically non-centrosymmetric material requires large, phase-pure, and reproducible single crystals, which in turn can only be obtained through a comprehensive exploration of the growth parameters and optimisation of the processing conditions. We have compared solid-state reactions, chemical vapour transport (CVT), and self-selecting vapour growth (SSVG) and have identified the key parameters that govern crystal quality and size. Building on the proven SSVG method for transition-metal halides,^30,31^ we applied this approach to RhSeCl, achieving large single crystals. Introducing SeCl_4_ as a new starting material further enhanced the chemical purity by eliminating RhCl_3_ intergrowths during crystal formation. By combining a two-step optimised SSVG process with the new reagent composition, we have substantially increased the final crystal size. Our results establish practical guidelines for producing high-quality RhSeCl crystals and provide a foundation for future physical properties studies and device applications of transition-metal Janus materials.

## 2 Experimental methods

### 2.1 Synthesis

As starting materials Rh powder (Alfa Aesar, 99.9%), RhCl_3_ powder (ChemPur, 49.9% Rh), Se pieces (Alfa Aesar, 99.999%), and SeCl_4_ powder (Sigma Aldrich, 35.0% Se) were used. All chemical handling was carried out inside an argon-filled glovebox due to the air-sensitivity of SeCl_4_ and RhCl_3_. All syntheses were performed in quartz ampoules with an inner diameter of 10 mm and wall thickness of 1 mm, sealed under dynamic vacuum, with the starting materials totalling a mass of approximately 200 mg. Two main combinations of starting materials were used to determine which would yield the best results. These were: (1) Rh : RhCl_3_ : Se with a molar ratio of 2 : 1 : 3 (with and without 10% of RhCl_3_ excess) and (2) Rh : SeCl_4_ : Se in a stoichiometric ratio of 4 : 1 : 3. The ampoules containing SeCl_4_ were initially cooled with ice and then with liquid nitrogen during the sealing of the ampoules to prevent the evaporation of SeCl_4_ and Se_2_Cl_2_ (details in results and discussion section). For crystal growth, two main approaches were applied:

#### 2.1.1. Chemical vapour transport method

The RhSeCl crystals were first grown by adapting the CVT protocol reported by Nowak *et al.*^27^ using the two different compositions of the starting materials described above. For all syntheses, 10 cm long quartz ampoules were used in a tubular furnace with a temperature difference of Δ*T* = 100 °C with *T*_1_ = 900 °C and *T*_2_ = 1000 °C.

#### 2.1.2. Self-selecting vapour growth method

The SSVG^30,31^ approach was adapted for RhSeCl using both reagent combinations. Two furnace configurations were tested: a box furnace mounted on its side and a tubular furnace (with one end of the quartz ampoule positioned in the middle of the furnace), both providing a very small temperature gradient. For all SSVG syntheses 6 cm long quartz ampoules were used. In the box furnace, the following temperature profile was applied: heating from room temperature (RT) with 50 °C h^−1^ up to 1000 °C, dwelling there for 24 h, then slowly cooling with −1 °C h^−1^ down to 900 °C, followed by cooling to RT at −150 °C h^−1^. For the syntheses in a tubular furnace, apart from the two reagent combination discussed above, we also performed recrystallization of RhSeCl (pre-synthesized by SSVG in a ‘flipped’ box furnace) and applied a similar temperature profile but at higher temperatures: heating from RT with 50 °C h^−1^ up to 1100 °C, dwelling there for 24 h, then slowly cooling with −1 °C h^−1^ down to 1075 °C, followed by cooling to RT at −150 °C h^−1^. In the tubular furnace, a temperature profile without slow cooling was also employed: heating from RT with 50 °C h^−1^ up to 1100, 1090, or 1075 °C, dwelling at the respective temperatures for 120 h, followed by cooling to RT with −150 °C h^−1^.

Furthermore, alternative SSVG syntheses were carried out using oscillating temperature profiles either between 1100 °C and 1000 °C or between 1000 °C and 900 °C, to probe possible crystal growth regions for the two reagent combinations described above. In both cases, the quartz ampoules were placed upright in alumina crucibles inside a conventional box furnace.

For the first variation (1100–1000 °C), the ampoule was heated from RT at 50 °C h^−1^ to 1100 °C, held for 24 h, and then cooled stepwise to 1075, 1050, 1025, 1000, and finally to 900 °C at a rate of −1 °C h^−1^, with each step including a 1 h dwell at the target temperature. The final cooling to RT was performed at −150 °C h^−1^. The second variation (1000–900 °C) followed the same principle. Theampoule was heated from RT at 50 °C h^−1^ to 1000 °C, held for 24 h, and then cooled stepwise to 975, 950, 925, and 900 °C, with 1 h dwell periods at each step. The sample was then cooled to RT at −150 °C h^−1^.

The grown crystals were washed under vacuum filtration with acetonitrile to remove any residues of Se_2_Cl_2_, then water to hydrolyse residues of SeCl_4_, and finally ethanol to dry the crystals. Any individual adaptations to these synthetic approaches, as well as further details and differences, are discussed in the results and discussion section.

### 2.2 Scanning electron microscopy and elemental analysis

The microstructure of the grown crystals was studied with a JEOL JSM-IT800 scanning electron microscope (SEM) equipped with an Oxford Silicon Drift Detector (SDD) X-Max^N^ that was used for the energy-dispersive X-ray spectroscopy (EDS) analysis (*U*_acc_ = 15–20 keV). Due to the semiconducting nature of RhSeCl, thin crystals were used to avoid charging, and larger crystals were cleaved with Kapton tape beforehand. For both the acquisition of the electron images and the collection of the EDS data, the crystals were fixed on a carbon pad or on a silicon wafer for the exfoliated flakes of RhSeCl.

### 2.3 Powder X-ray diffraction

Powder X-ray diffraction (PXRD) data (Cu-K_α_, *λ* ≈ 154 pm, RT) were collected on a Rigaku SmartLab diffractometer (Rigaku, Debye–Scherrer geometry, D/tex detector). The crystals for the measurements were finely ground with corn starch in an agate mortar to decrease the preferred orientation of the strongly layered material. The powders were then sealed in borosilicate glass capillaries (Hilgenberg GmbH, outer diameter *∅* = 0.8 mm, wall thickness of 0.01 mm). Rietveld refinements were performed using TOPAS Academic software.^32^

### 2.4 Single crystal X-ray diffraction

Single crystal X-ray diffraction (SCXRD) was measured on a four-circle Oxford Diffraction SuperNova diffractometer (Agilent/Rigaku SE) with mirror optics and an Atlas CCD detector at *T* = 100(1) °C or *T* = 250(1) °C. Mo-K_α_ (*λ* = 71.073 pm) was used. The crystals were prepared in immersion oil and mounted using Cryoloops. A numerical (Gaussian) absorption correction based on the crystal shape was applied, and the structure was solved with direct methods and subsequent refinements against *F*^2^_o_ using SHELXL^33,34^ and Olex2.^35^ The integrated CrysAlisPro software was used for data collection and initial treatment of the data.^36^

### 2.5 Exfoliation and Raman spectroscopy

The RhSeCl crystals were exfoliated using standard adhesive-tape methods. Thin flakes were transferred to Si/SiO_2_ substrates (285 nm oxide) and examined under an optical microscope to identify suitable regions for Raman measurements. The number of layers was determined by the optical contrast of the flakes relative to the substrate, following established calibration methods. Raman spectra were recorded with a commercial confocal Raman microscope HORIBA LabRAM HR Evolution equipped with a 532 nm continuous-wave laser. A circularly polarised laser beam was focused through an Olympus ×100 (NA = 0.9) objective onto the sample, forming a spot with FWHM ∼ 0.3 μm. To avoid overheating, the laser power at the sample was reduced to 200 μW, as confirmed by temperature estimates in the laser spot derived from the Stokes/anti-Stokes ratio.

## 3 Results and discussion

In our exploratory work on the preparation of large RhSeCl crystals, we performed a comparative study of different synthesis and growth methods. In the following, we will compare the reactions that develop from two different combinations of starting materials, one based on RhCl_3_ (Rh : RhCl_3_ : Se = 2 : 1 : 3) and described by eqn (2), another using SeCl_4_ (Rh : SeCl_4_ : Se = 4 : 1 : 3) and described by eqn (3).

3 4Rh + 3Se + SeCl_4_ = 4RhSeCl

The use of SeCl_4_ as an alternative chlorine source provides a new synthetic pathway for RhSeCl, enabling a direct comparison of how precursor chemistry influences crystal growth. We employed both reactions with all processing methods investigated here, which are solid-state reaction, chemical vapour transport (CVT), and self-selecting vapour growth (SSVG). These experiments established a baseline for assessing which approach offers the most reliable route to large, phase-pure single crystals.

In the following, we will refer to the first reagent combination as ‘RhCl_3_’ and the second as ‘SeCl_4_’. It is important to note that when SeCl_4_ is used as a starting material, the following reaction occurs at room temperature as soon as Se and SeCl_4_ are mixed together in a quartz ampoule:

4 SeCl_4_ + 3Se → 2Se_2_Cl_2_

This could be visually observed through the formation of a brown-red liquid.

### 3.1 Chemical vapour transport crystal growth

**Fig. 1:**
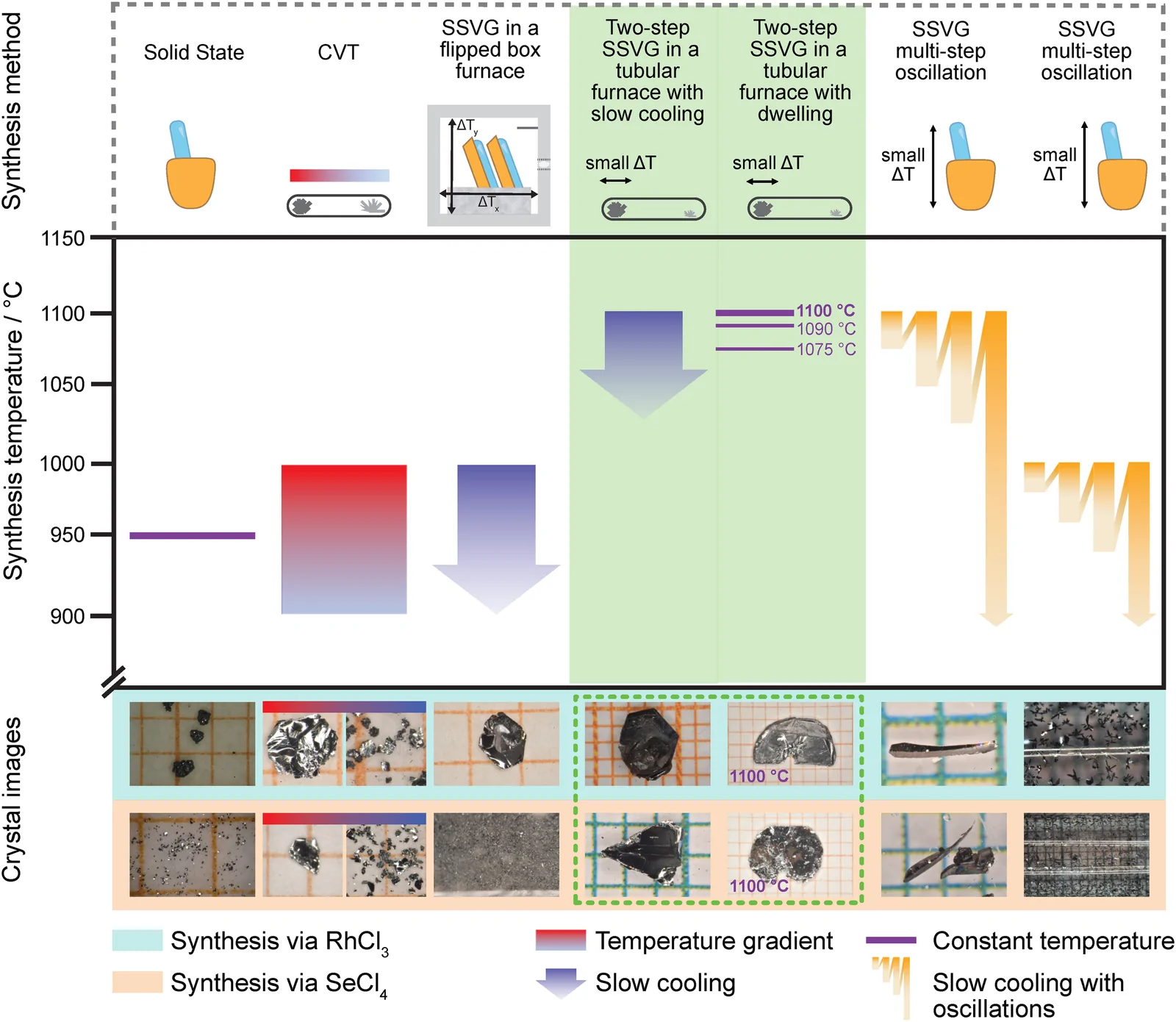
Synthesis map for RhSeCl crystal growth. Investigated methods are shown in the upper panel. Temperature profiles for each technique are presented in the middle. Pictures of the RhSeCl crystals taken with an optical microscope on millimetre paper at the bottom correspond to the synthesis method above. The turquoise and orange backgrounds represent different synthesis paths: *via* Rh, Se, RhCl_3_ or Rh, Se, SeCl_4_ reagent combinations, respectively. For the SSVG in a tubular furnace, the shown crystals are obtained by two-step SSVG process starting from RhCl_3_ and SeCl_4_, with the first step being SSVG in a ‘flipped’ box furnace. The green background and the dashed frame highlight the best synthesis methods and conditions, which allow the growth of the largest RhSeCl crystals.

**Fig. 2:**
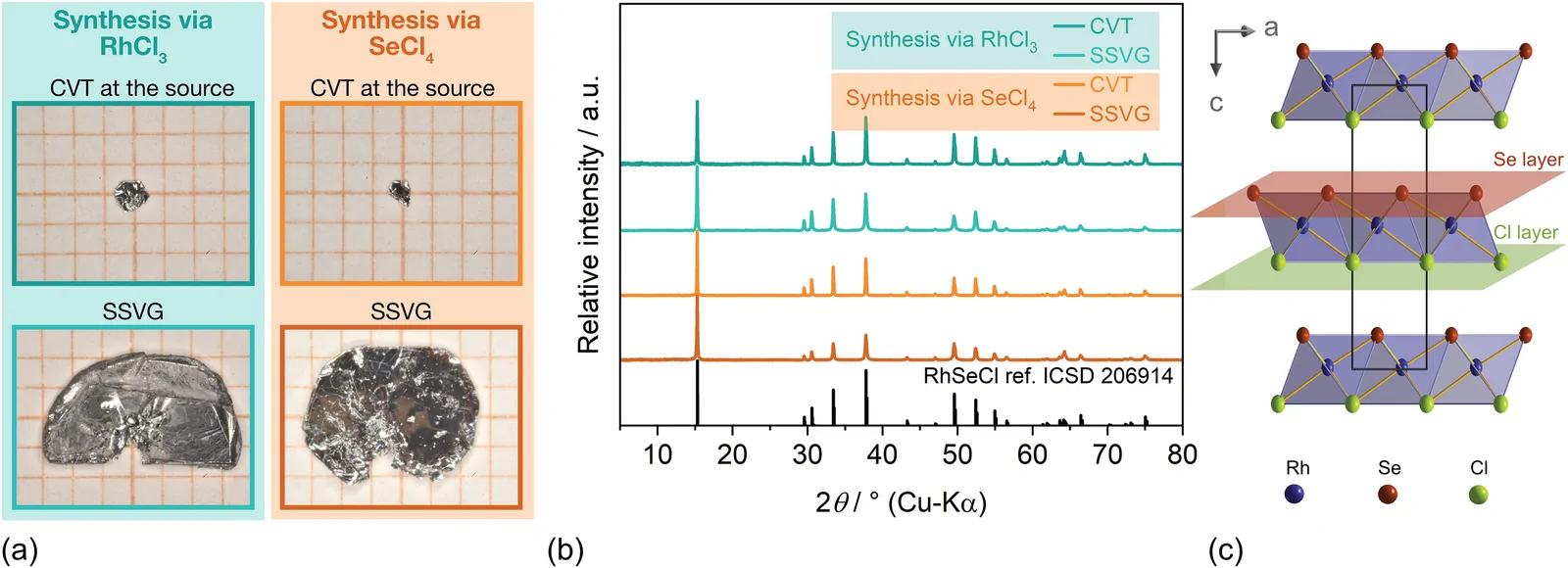
Grown crystals of RhSeCl and crystal structure. (a) Comparison of the RhSeCl crystals grown by chemical vapour transport (CVT) and self-selecting vapour growth (SSVG). The crystals grown with RhCl_3_ are highlighted *via* a turquoise background, while the crystals grown with SeCl_4_ are highlighted *via* an orange background. (b) PXRD patterns of washed crystals from the different types of reagent combinations and synthesis types. The crystals grown from RhCl_3_ are in turquoise, the crystals grown with SeCl_4_ are in orange, and the RhSeCl reference (ICSD 206914) is in black. (c) The crystal structure from a refinement of a RhSeCl crystal grown *via* the two-step SSVG synthesis with RhCl_3_ as a reagent. The structure here is viewed along the crystallographic *b*-axis to highlight the ordered Janus phase with the Cl atoms forming one layer on one side of the central Rh and the Se atoms forming a layer on the other side.

To establish a reference point for crystal quality, we first reproduced the CVT syntheses reported earlier, using, as discussed above, the previously employed RhCl_3_, as well as the here introduced SeCl_4_-based precursor mixtures. In both cases, the reactions yielded high phase purity RhSeCl single crystals with comparable morphologies. Interestingly, crystal formation occurred at both ends of the ampoules. At the sink (*T*_1_ = 900 °C), we observed intergrown crystal aggregates similar to those previously described,^27^ whereas the source (*T*_2_ = 1000 °C) contained larger crystals that reached approximately 1 mm across (Fig. 1 and 2a). This observation suggests that maintaining a small temperature gradient near the solid source promotes crystal growth, a condition inherently realised in the SSVG method. In contrast, under a large temperature gradient of CVT nucleation was not under control, as a consequence, the crystals could not grow large in size. Encouraged by this and by the earlier success of SSVG in the Co_1−*x*_Ni_*x*_I_2_, RuCl_3_, and other transition metal halide systems,^30,31^ we next investigated SSVG as an independent growth technique for RhSeCl.

### 3.2 Self-selecting vapour growth

To test whether SSVG alone could promote RhSeCl crystal growth, we next performed experiments in a ‘flipped’ box furnace, positioned on its side.^30,31^ Ampoules containing RhCl_3_- or SeCl_4_-based precursor mixtures were heated to 1000 °C, held for 24 h and then slowly cooled to 900 °C at a rate of −1 °C h^−1^ before returning to room temperature. Indeed, crystals with lateral size up to 1 mm formed when the route with RhCl_3_ was used and were comparable in size to those obtained by CVT at the source. For the route with SeCl_4_ predominantly phase-pure polycrystalline powder was observed (Fig. 1). These results demonstrate that SSVG can be utilised to synthesise RhSeCl single crystals.

### 3.3 Crystal growth optimization

Since we succeeded in growing small RhSeCl crystals *via* SSVG technique in the ‘flipped’ furnace, we then explored whether higher temperatures and a different temperature gradient could further enhance growth. To achieve this, we carried out SSVG in short ampoules (6 cm), with the end containing the precursors placed in the centre of a one-zone tubular furnace. This provided a small thermal gradient without inducing undesired CVT transport. We tested three precursor systems: RhCl_3_-, SeCl_4_-based mixtures, and polycrystalline RhSeCl pre-synthesised *via* SSVG in a ‘flipped’ furnace (without opening the ampoule between the syntheses). These experiments were carried out in a similar temperature profile as for the previously outlined SSVG setup, but the temperatures were increased to 1100–1075 °C. Under these conditions, all reactions produced larger crystals (2–4 mm), and the best results were obtained with pre-synthesised *via* SSVG in the ‘flipped’ furnace RhSeCl as the starting material (Fig. 1). These findings showed that higher growth temperatures promote crystal enlargement, establishing a two-step SSVG route – initial polycrystalline formation followed by regrowth in a tubular furnace – as the most effective strategy for producing large RhSeCl crystals.

Building on the observation that higher growth temperatures improved crystal size, we next optimised the two-step SSVG synthesis by introducing an isothermal dwelling step in the tubular furnace. Using pre-synthesised in a ‘flipped’ furnace RhSeCl as starting material, we examined the dwell temperatures of 1075, 1090, and 1100 °C with a duration of 120 h. The 1100 °C annealing proved to be the most effective, yielding the largest crystals up to 0.4 × 5 × 6 mm (Fig. 1 and 2), while even at lower temperatures, crystals exceeding 2 mm were readily obtained. Maintaining a closed ampoule between the two synthesis steps was essential: reopening or washing the intermediate product led to incongruent melting and decomposition into RhSe, Rh, and SeCl_4_ (see SI). This is likely because sealed tubes retain volatile Cl and Se-bearing species that act as transport agents during crystal growth. Attempts at higher dwell temperatures (>1100 °C) again produced incongruent melts. Large crystals grown under the best conditions often exhibited a small cavity at the base, suggesting growth from a partially molten phase. Altogether, these experiments identify 1100 °C as the optimal temperature for reproducible growth of large RhSeCl crystals *via* the two-step SSVG approach.

### 3.4 Self-selecting vapour growth for crystals with needlelike morphology

As a final set of experiments, we decided to see whether SSVG reactions where the temperature was oscillated in the previously determined crystal growth regions would lead to any significant improvement or change in the crystal growth. In this case, we carried out the experiments as outlined in the experimental section above, oscillating the temperature either between 1100 and 1000 °C or between 1000 and 900 °C. In both cases, the main difference in comparison to all the other synthetic approaches investigated here lies in the morphology of the grown crystals. While the size of individual crystals was comparable to previously reported results, the crystals grew preferentially in one direction, taking on a needle-like appearance, as can be seen in the last two columns of Fig. 1. Interestingly, crystal growth was also not localised at one end of the ampoule, but was rather consistent along the walls, indicating that there was constant movement and deposition of RhSeCl crystal nuclei through the temperature oscillations, demonstrating an SSVG type of growth in a natural vertical temperature gradient of a box furnace. Despite their different morphology, these crystals were found to be structurally identical to the other RhSeCl crystals obtained, as proven by SCXRD measurements (Tables S1 and S6).

### 3.5 Comparison of the crystal growth methods

Comparing all crystal-growth approaches reveals how both the reaction chemistry and the thermal environment govern the formation of high-quality RhSeCl crystals. Conventional CVT methods, as previously reported in the literature, reliably produces RhSeCl crystals, however, their size remains limited. CVT growth for RhSeCl occurs primarily through vapour transport between the hot and cold ends of the ampoule, leading to agglomerates of small crystallites, while additional evaporation–condensation processes at the source can occasionally yield larger, isolated crystals. In contrast, the SSVG technique in a ‘flipped’ box furnace enables growth directly within a minimal temperature gradient, and when applied using RhCl_3_-based precursors, it yields crystals comparable in size to those obtained in CVT reactions at the source.

Increasing the temperature and changing the temperature gradient within short ampoules in a tubular furnace markedly improved the crystal size, indicating that crystal growth in this system is favoured by higher vapour pressures and a partially molten phase. The most significant improvement in RhSeCl growth was achieved in the two-step SSVG process: first generating polycrystalline RhSeCl *via* SSVG in a ‘flipped’ furnace and then regrowing crystals at elevated temperature in a tubular furnace. Using this two-step process, we could grow RhSeCl crystals up to 6 mm in lateral size for the synthesis with a dwelling step at 1100 °C. Maintaining a sealed environment between steps was essential: opening the ampoules disrupted the internal vapour balance and led to incongruent melting.

Taken together, these results demonstrate that successful RhSeCl crystal growth depends on ensuring an optimal combination of high temperature, limited temperature gradient, and closed-system vapour chemistry. The two-step SSVG approach thus combines the advantages of solid-state synthesis and vapour transport, providing a reproducible pathway to large, phase-pure RhSeCl single crystals suitable for further physical characterisation and device fabrication. The overall trends and optimal conditions are summarised in the synthesis map (Fig. 1), with alternative routes detailed in the SI.

### 3.6 Crystal structure

Powder X-ray diffraction of all our washed samples predominantly showcased the presence of the phase-pure RhSeCl Janus phase. Rietveld refinements of the diffraction patterns obtained for each synthetic approach confirmed an excellent fit to the previously reported structure model of RhSeCl, *i.e.*, for the samples grown with SeCl_4_ from our two-step SSVG approach we obtained cell parameters of *a* = 348.4(2) pm and *c* = 1156.9(7) pm in the space group *P*6_3_*mc*. PXRD patterns for the CVT-grown crystals and for the largest RhSeCl crystals produced in this work are shown in Fig. 2, while the data for all other syntheses, together with the Rietveld analyses, are presented in the SI.

We tested the single-crystals of the individual methods by SCXRD to verify the crystal quality, and we found all of them to crystallise in the hexagonal space group *P*6_3_*mc*. The crystals grown by the herein newly reported SSVG approach with SeCl_4_ as a precursor have lattice parameters of *a* = 349.11(1) pm and *c* = 1158.89(3) pm, while the single-crystals grown from SeCl_4_ that take on a needle-like morphology (see section 3.4) show lattice parameters of *a* = 349.15(3) pm and *c* = 1158.29(12) pm. In both cases, these parameters vary merely by approximately 0.1% from those found in earlier work and hence no significant differences in the crystal structures could be observed (see Table S2).

As expected for these Janus structures, as seen in a representative crystal that was grown from SeCl_4_ with the two-step SSVG method, the central Rh atom is coordinated by three Cl atoms with a distance of 250.84(23) pm and by three Se atoms with a distance of 240.34(8) pm in the expected facial arrangement with the anions ordering according to their type on opposite sides (see Fig. 2). These bond lengths only vary minimally from the previously recorded crystal structure of RhSeCl (<0.1%). Furthermore, the residual electron density peaks fall within the range of ±1 to 2 electrons ×10^−6^ pm^−3^, mostly within 0.5–2 Å of Rh, or 0.5–1.5 Å of Se. The higher residual electron density peaks near the metal cation (Rh) are expected, especially considering the size of Rh. Although the peaks are close to Rh and Se, they do not indicate disorder, as they are below 5 electrons ×10^−6^ pm^−3^ and no negative density is observed on the atomic sites. Additionally, the Debye–Waller factors remain reasonable at both 250 and 100 K (see Table S2), and also do not indicate any substitutional or dynamic disorder.

We also investigated the possibility of any substitutional disorder by refining mixed Se and Cl sites on both atomic positions. However, this resulted in no significant change in the occupancy of the ‘original’ atom on that position: in the case of the Cl position, *i.e.*, the 2*a* Wyckhoff position, the occupancy remained 99.5 to 99.8% Cl and on the Se position, *i.e.*, the 2*b* Wyckhoff position, the occupancy remained approx. 99.5% Se, and in both cases, there was no significant change in the refinement parameters. All of this further confirms that there is very little to no intermixing of the Se and Cl sites, and that the Janus structure remains.

And finally, we also checked whether any twinning could be observed. First, the possibility of non-merohedral twinning was examined, however, here we found that at most 5% of the reflections could be assigned to a further twin component. Due to the small presence of the twinned component and the significant negative impact on the structure solutions where a twin component was present, we did not include it in the final structure solution, especially as other warning signs of non-merohedral twinning, *i.e.*, the *F*_o_ values of the most disagreeable reflections are not significantly larger than the *F*_c_ values, nor is the *K* = mean(*F*^2^_o_)/mean(*F*^2^_c_) systematically high for the low-intensity reflections, were not present.

And finally, the possibility of twinning by merohedry was also investigated, *e.g.*, in the crystal grown *via* the CVT method from SeCl_4_ had a potential merohedric twin with a BASF value of 0.02(4) and a twin law of (−1 0 0|1 1 0|0 0 −1). However, when this is taken into account in the refinement of the structure solution, it has next to no effect on the refinement parameters, which also indicates that there is no significant twinning by merohedry. All of this together indicates that despite small, individual crystal-dependent factors, all of the synthetic methods that we have outlined here produce high-quality single crystals.

### 3.7 Exfoliation of RhSeCl

One of the initial aims of producing larger crystals was to investigate their viability in device applications. As reported in both previous works^27,29^ and confirmed here by our CVT-grown crystals, conventional vapour transport only provides small crystals at the lower limit of what is required in terms of exfoliation of thin flakes. Following the successful growth of larger crystals *via* the two-step SSVG method, we began investigations to determine whether these crystals of RhSeCl were also suitable for potential device fabrication. We could successfully exfoliate the RhSeCl van der Waals material using the standard mechanical scotch-tape method. We reproducibly obtained large flakes down to few-layers and monolayer thicknesses, which indicates that the crystals grown *via* the two-step SSVG route are indeed suitable for Janus-type device fabrications. To assess the structural and chemical uniformity of these exfoliated layers, we examined both bulk and few-layer flakes using EDX and Raman spectroscopy.

**Fig. 3:**
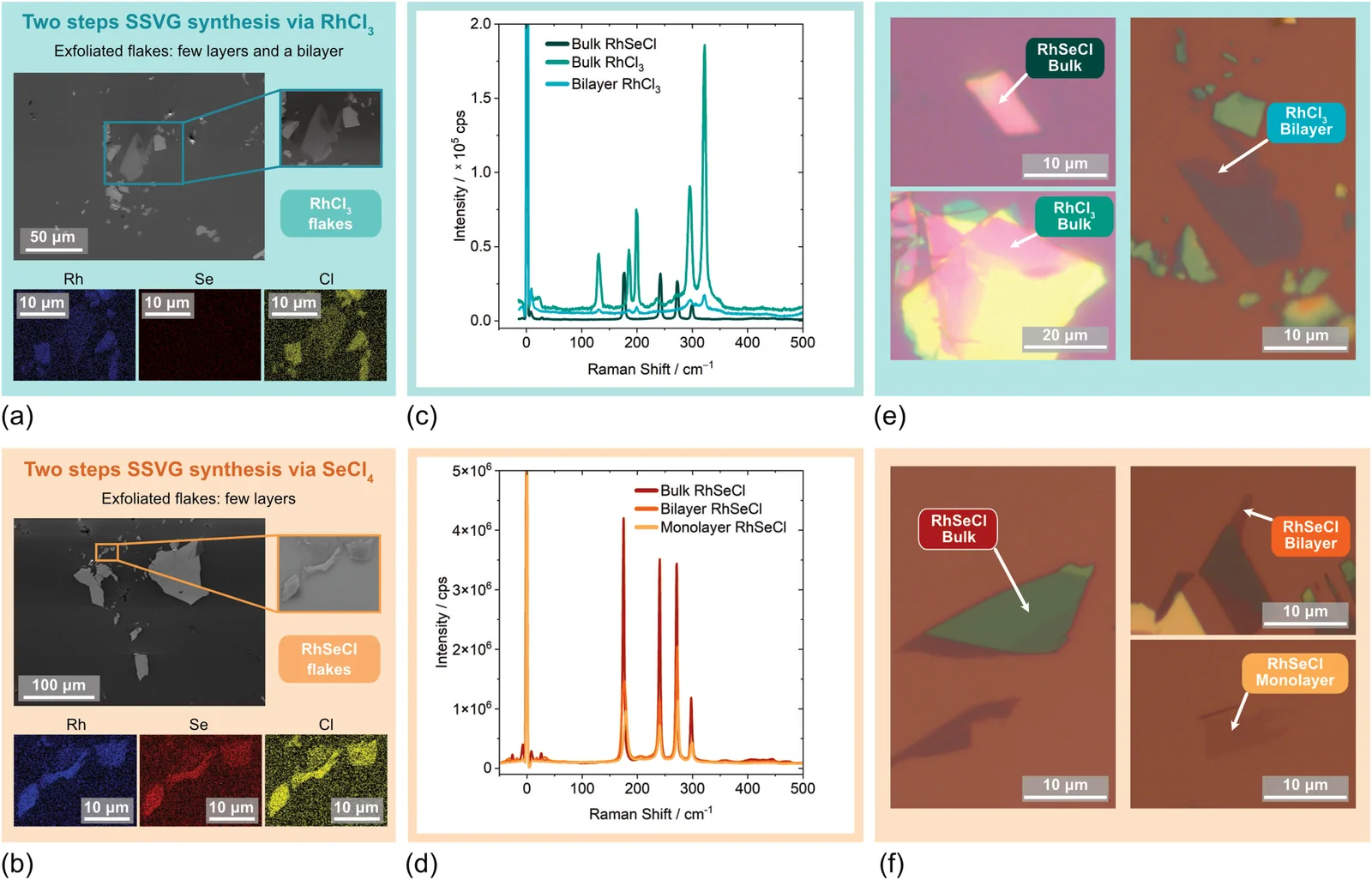
Elemental analysis and Raman spectra of bulk and exfoliated RhSeCl crystals grown *via* two-step SSVG starting from two different reagent combinations. The upper panels show results for RhSeCl crystals grown from RhCl_3_, the bottom ones from SeCl_4_. (a and b) Micrograph and elemental analysis of exfoliated RhSeCl crystals. The dominant phase observed is RhCl_3_ for crystals grown from RhCl_3_, while for crystals grown from SeCl_4_ it is RhSeCl. (c and d) Comparison of Raman spectra of bulk and exfoliated RhSeCl crystals. (e and f) Polarised optical microscope pictures of RhSeCl flakes on which Raman measurements were performed.

For crystals with RhCl_3_ as the starting reagent in the two-step SSVG synthesis, we observed two different phases with Raman spectroscopy (see Fig. 3) after being exfoliated. We found that these RhSeCl crystals in fact contain some intergrown layers of RhCl_3_ which only became visible once we began to exfoliate the crystals down to few-layers (bulk crystals were checked by PXRD, SCXRD and EDX prior to exfoliation, see SI). We confirmed this through EDS analysis of the same bilayers and bulk flakes, as can be seen in Fig. 3(a).

This impurity becomes visible after exfoliation, probably because RhCl_3_ exfoliates more easily than RhSeCl, and due to this, even tiny amounts of intergrown RhCl_3_ result in the dominant appearance of RhCl_3_ flakes.

When we repeated the same exfoliation experiments with the crystals grown starting from SeCl_4_, we observed that they did not contain any inclusions of RhCl_3_, as can be seen in Fig. 3(b), (d) and (f). Even down to the monolayer, only pure RhSeCl was found, which was reproducible between all batches grown starting from SeCl_4_*via* the two-step SSVG synthesis route. Hence, the crystals grown as such are the only ones that are both large enough to be used in device applications and for exfoliation experiments, and in addition to this, they are free of inherently intergrown impurities that would hinder their suitability.

As a result of both the optimizations in crystal synthesis with regard to the method and the combination of reagents, together with the initial EDX and Raman investigations of the exfoliated bilayer and few-layer flakes, we can say that the crystals grown from SeCl_4_ by the two-step SSVG method yield the best, reproducible and most promising samples for further explorations towards device fabrication.

## 4 Conclusions

We have thoroughly investigated the crystal growth process of the recently discovered and highly promising Janus material RhSeCl. We grew single crystals using various methods and two different reagent combinations with RhCl_3_ and SeCl_4_ as the chlorine source. First, we could confirm and reproduce the results previously obtained by the CVT method and proved that the crystal size remains very limited while using this technique. We found that the two-step SSVG process (pre-synthesis performed in a ‘flipped’ furnace, followed by recrystallisation at higher temperatures in a tubular furnace) provides reproducible crystals up to 0.4 × 5 × 6 mm^3^. Moreover, the choice of the precursor phases was also found to be critical. We observed the intergrowth of thin RhCl_3_ flakes when this compound is used as a reagent. The volume fraction of these RhCl_3_ flakes was not visible by bulk-sensitive probes, but was sufficient to dramatically affect the device fabrication. We propose an alternative synthesis approach in which SeCl_4_ is used as a precursor, and we proved that in this case, no intergrown impurities were detected and the exfoliated flakes all show a high and reproducible quality. Taken together, we find the optimal crystal growth conditions to be a two-step SSVG process, starting with the synthesis of polycrystalline RhSeCl from Rh, Se, and SeCl_4_ in a ‘flipped’ furnace *via* slow cooling from 1000 °C to 900 °C, followed by recrystallisation by dwelling at 1100 °C (or slow cooling from 1100 °C to 1075 °C) in a tubular furnace to yield large single crystals. These optimised conditions now provide reliable access to large, structurally pure RhSeCl crystals, creating a solid foundation for meaningful device-level studies.

## Conflicts of interest

There are no conflicts to declare.

## Supplementary Material

CE-028-D5CE01170A-s001

## Data Availability

The data supporting this article have been included as part of the supplementary information (SI). Supplementary information is available. See DOI: https://doi.org/10.1039/d5ce01170a.

## References

[cit1] Kageyama H., Hayashi K., Maeda K., Attfield J. P., Hiroi Z., Rondinelli J. M., Poeppelmeier K. R. (2018). Nat. Commun..

[cit2] Harada J. K., Charles N., Poeppelmeier K. R., Rondinelli J. M. (2019). Adv. Mater..

[cit3] Oka D., Hirose Y., Kamisaka H., Fukumura T., Sasa K., Ishii S., Matsuzaki H., Sato Y., Ikuhara Y., Hasegawa T. (2014). Sci. Rep..

[cit4] Ishizaka K., Bahramy M., Murakawa H., Sakano M., Shimojima T., Sonobe T., Koizumi K., Shin S., Miyahara H., Kimura A. (2011). et al.. Nat. Mater..

[cit5] Zhang Y., Wu H., Hu Z., Yu H. (2023). Chem. – Eur. J..

[cit6] Gibson Q. D., Manning T. D., Zanella M., Zhao T., Murgatroyd P. A., Robertson C. M., Jones L. A., McBride F., Raval R., Cora F. (2019). et al.. J. Am. Chem. Soc..

[cit7] Ziebel M. E., Feuer M. L., Cox J., Zhu X., Dean C. R., Roy X. (2024). Nano Lett..

[cit8] Wu F., Gutierrez-Lezama I., Lopez-Paz S. A., Gibertini M., Watanabe K., Taniguchi T., von Rohr F. O., Ubrig N., Morpurgo A. F. (2022). Adv. Mater..

[cit9] Lopez-Paz S. A., Guguchia Z., Pomjakushin V. Y., Witteveen C., Cervellino A., Luetkens H., Casati N., Morpurgo A. F., von Rohr F. O. (2022). Nat. Commun..

[cit10] Lee J., Ko T. Y., Kim J. H., Bark H., Kang B., Jung S.-G., Park T., Lee Z., Ryu S., Lee C. (2017). ACS Nano.

[cit11] Wang Y.-X., Graham T. K., Rama-Eiroa R., Islam M. A., Badarneh M. H., Nunes Gontijo R., Tiwari G. P., Adhikari T., Zhang X.-Y., Watanabe K. (2025). et al.. Nat. Mater..

[cit12] Cheng Y., Zhu Z., Tahir M., Schwingenschlogl U. (2013). Europhys. Lett..

[cit13] Lu A.-Y., Zhu H., Xiao J., Chuu C.-P., Han Y., Chiu M.-H., Cheng C.-C., Yang C.-W., Wei K.-H., Yang Y. (2017). et al.. Nat. Nanotechnol..

[cit14] Zhang L., Yang Z., Gong T., Pan R., Wang H., Guo Z., Zhang H., Fu X. (2020). J. Mater. Chem. A.

[cit15] Zhang L., Yu J., Yang M., Xie Q., Peng H., Liu Z. (2013). Nat. Commun..

[cit16] Bissett M. A., Takesaki Y., Tsuji M., Ago H. (2014). RSC Adv..

[cit17] Li R., Cheng Y., Huang W. (2018). Small.

[cit18] Zhang J., Jia S., Kholmanov I., Dong L., Er D., Chen W., Guo H., Jin Z., Shenoy V. B., Shi L., Lou J. (2017). ACS Nano.

[cit19] Liu M., Wu W., Chen Z., Zhang Y., Yu X., Yang S., Wang H., Xu F., Chen L., Li X. (2025). et al.. Nano Lett..

[cit20] Hajra D., Sailus R., Blei M., Yumigeta K., Shen Y., Tongay S. (2020). ACS Nano.

[cit21] Sklyadneva I. Y., Heid R., Bohnen K.-P., Chis V., Volodin V., Kokh K., Tereshchenko O., Echenique P., Chulkov E. (2012). Phys. Rev. B: Condens. Matter Mater. Phys..

[cit22] Jacimovic J., Mettan X., Pisoni A., Gaal R., Katrych S., Demko L., Akrap A., Forro L., Berger H., Bugnon P., Magrez A. (2014). Scr. Mater..

[cit23] Akrap A., Teyssier J., Magrez A., Bugnon P., Berger H., Kuzmenko A. B., van der Marel D. (2014). Phys. Rev. B: Condens. Matter Mater. Phys..

[cit24] Wang J., Yuan X., Fang Y., Chen X., Zhong Z., Lin S., Qu J., Fu J., Liu Y., Li Z. (2025). et al.. Small.

[cit25] Yun J. H., Sung M., Choi M., Kim K., Yang W., Kim D., Kim M. J., Her S.-H., Choi S.-Y., Kim T.-H. (2025). et al.. Adv. Mater..

[cit26] Kretschmer J. , PhD thesis, Rheinische Friedrich-Wilhelms-Universitat Bonn, 2019

[cit27] Nowak D., Valldor M., Rubrecht B., Froeschke S., Eltoukhy S., Buchner B., Hampel S., Grasler N. (2023). Inorg. Chem. Front..

[cit28] Nowak D. , PhD thesis, Technische Universität Dresden, Dresden, 2025

[cit29] Liu K., Sun X., Cheng P., Li Z., Li P., Jia D., Zhao S., Yang X., Wang X., Ye L. (2025). et al.. Adv. Sci..

[cit30] Yan J.-Q., McGuire M. A. (2023). Phys. Rev. Mater..

[cit31] Lukovkina A., Lopez-Paz S. A., Besnard C., Guenee L., von Rohr F. O., Giannini E. (2024). Chem. Mater..

[cit32] Coelho A. A. (2018). J. Appl. Crystallogr..

[cit33] Sheldrick G. M. (2015). Acta Crystallogr., Sect. A: Found. Adv..

[cit34] Sheldrick G. M. (2015). Acta Crystallogr., Sect. C: Struct. Chem..

[cit35] Dolomanov O. V., Bourhis L. J., Gildea R. J., Howard J. A., Puschmann H. (2009). J. Appl. Crystallogr..

[cit36] CrysAlis PRO, Agilent Technologies Ltd., Yarnton, Oxfordshire, England, 2014

