# Supplementary material for: Growth of large crystals of Janus phase RhSeCl using self-selecting vapour growth
Source: CrystEngComm. 2026 Jan 30;28(7):1256–64. doi: 10.1039/d5ce01170a (PMC12857252; doi:10.1039/d5ce01170a)
Supplement: CE-028-D5CE01170A-s001 [file CE-028-D5CE01170A-s001.pdf]

Supplementary Information (SI) for CrystEngComm.
This journal is © The Royal Society of Chemistry 2026

Supporting Information: Growth of Large Crystals of Janus Phase RhSeCl Using Self-Selecting Vapor Growth

Anastasiia Lukovkina‡, Maria A. Herz‡, Xiaohanwen Lin, Volodymyr Multian, Alberto Morpurgo, Enrico Giannini, and Fabian O. von Rohr*

Department of Quantum Matter Physics, University of Geneva, CH-1211 Geneva, Switzerland

E-mail: fabian.vonrohr@unige.ch

Solid-State Synthesis

We additionally repeated the initial solid-state experiments from *Nowak et al.*, using two reagent combinations: Rh : $RhCl_3$: Se with a ratio of 2 : 1 : 3 and Rh : $SeCl_4$: Se in a stoichiometric ratio of 4 : 1 : 3. We performed the reaction at 950 °C for 120 h. We washed the polycrystalline agglomerate as per the protocol in the Experiment section and observed the formation of RhSeCl, which we confirmed by PXRD as seen in Fig. 1 and Fig. 2. This method resulted in small crystals, shown in the Fig. 1, Column 1 of the main paper. These initial experiments confirmed that the use of $SeCl_4$ as a starting reagent was a comparable alternative to using a combination with $RhCl_3$, and we continued to apply it in all of our following experiments.

Powder X-ray Diffraction

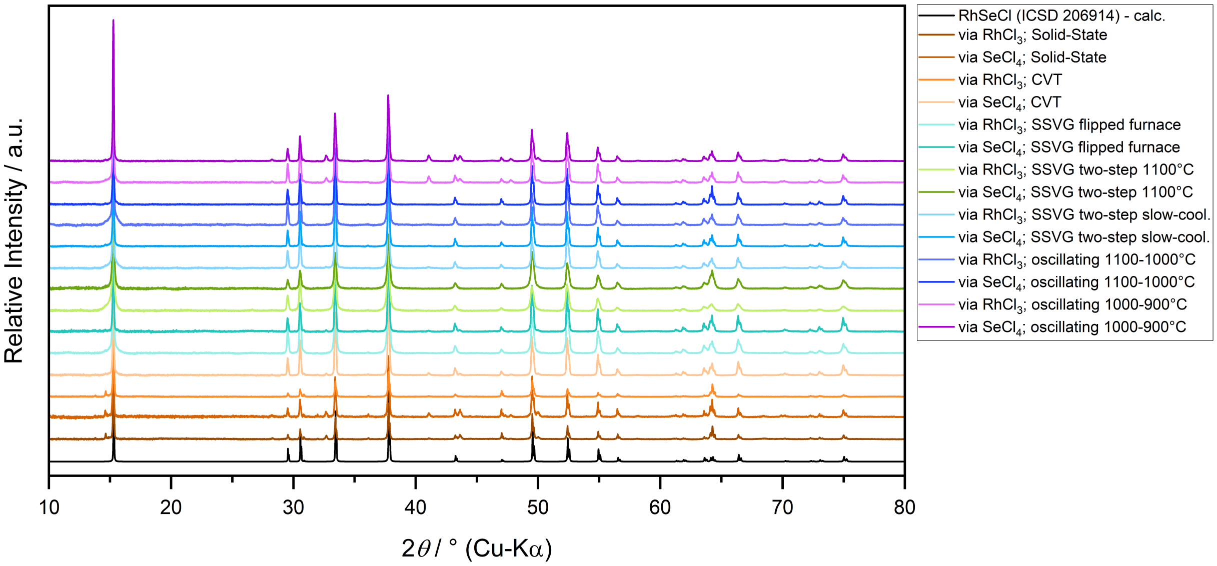

Figure 1: PXRD patterns of all of the different synthetic routes investigated. The black pattern represents the reference pattern of RhSeCl from the ICSD. The colored patterns represent the experimentally measured data. The backgrounds of all experimental patterns have been evened out in the software Match! through subtraction of the capillary and starch reference background to ensure an easier comparison of experimental patterns.

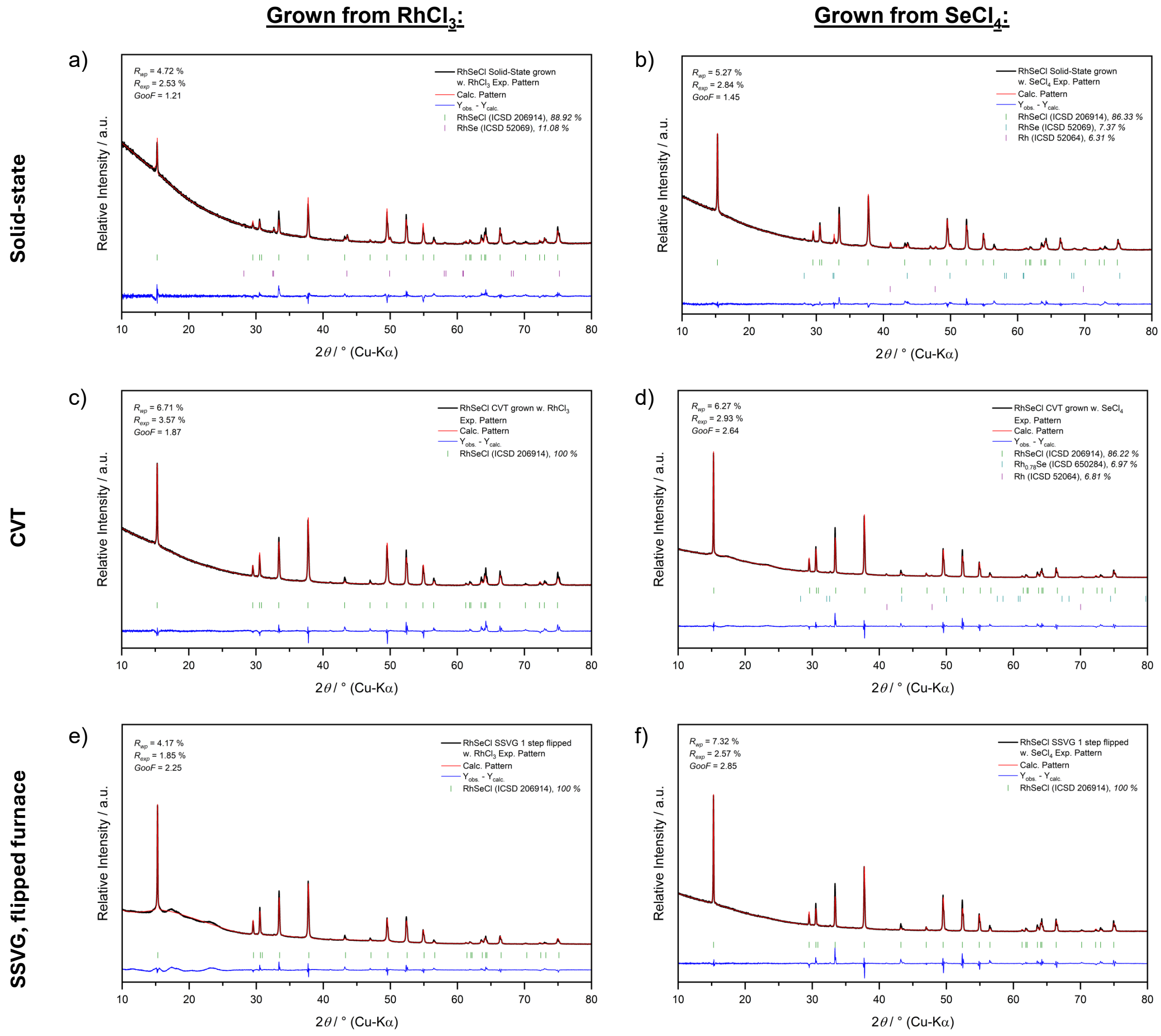

Figure 2: Rietveld analysis of RhSeCl polycrystalline powder grown via: (a) and (b) the solid-state method; (c) and (d) the CVT method; and (e) and (f) the SSVG method with one step in the 'flipped' furnaces.

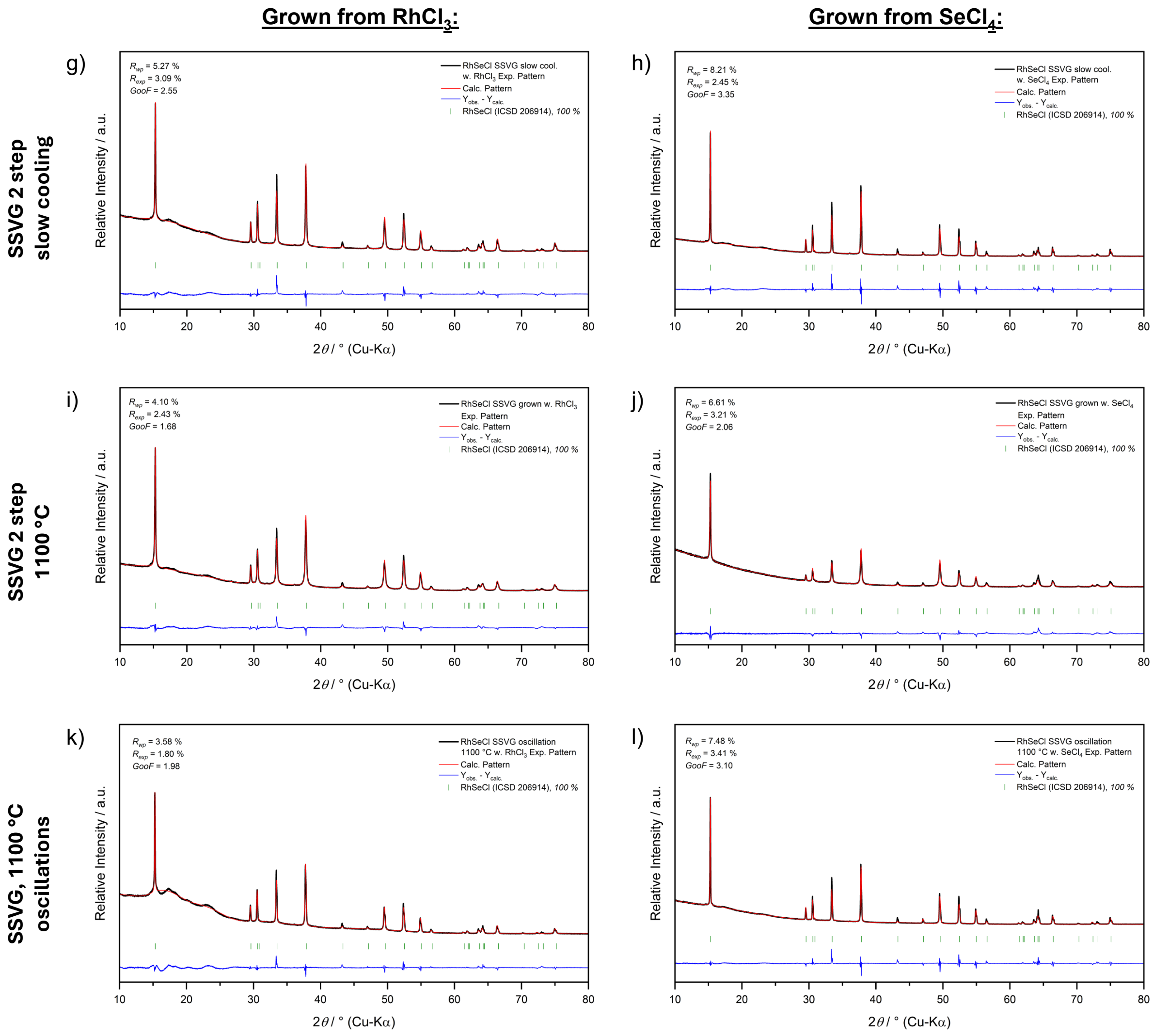

Figure 3: Rietveld analysis of RhSeCl polycrystalline powder grown via: (g) and (h) the two-step SSVG method including the slow cooling in the second step; (i) and (j) the two-step SSVG method with annealing at 1100 °C in the second step; and (k) and (l) the oscillating SSVG method starting from 1100 °C.

Table 1: Parameters of the Rietveld refinements of all investigated synthesis methods (Fig. 2 and Fig. 3). The samples marked with * contain RhSe and Rh impurities, which were all refined separately. All other refinements represent only one RhSeCl phase.

**Synthesis Route**	**RhSeCl** $a = b$ pm	c pm	$\alpha = \beta$ °	γ °	**Figures of Merit** R_{wp} %	R_p %	R_{exp} %	*GooF*
Solid state ($RhCl_3$)*	349.0(7)	1158.8(2)	90	120	4.72	2.53	3.90	1.21
Solid state ($SeCl_4$)*	349.4(1)	1160.2(4)	90	120	5.27	2.84	3.64	1.45
CVT ($RhCl_3$)* *at the source*	348.7(2)	1157.8(9)	90	120	6.71	3.18	3.57	1.88
CVT ($SeCl_4$)* *at the source*	348.7(1)	1158.0(4)	90	120	6.27	2.93	2.37	2.64
SSVG ($RhCl_3$) *'flipped' furnace*	348.3(1)	1156.4(4)	90	120	4.17	2.75	1.85	2.25
SSVG ($SeCl_4$) *'flipped' furnace*	348.9(1)	1158.9(5)	90	120	7.32	3.24	2.57	2.85
SSVG ($RhCl_3$) *2 step slow cooling*	348.1(1)	1155.9(4)	90	120	5.27	3.09	2.07	2.55
SSVG ($SeCl_4$) *2 step slow cooling*	348.6(1)	1157.3(4)	90	120	8.21	4.30	2.45	3.35
SSVG ($RhCl_3$) *2 step* 1100 °C	347.7(1)	1155.1(3)	90	120	4.09	2.86	2.43	1.68
SSVG ($SeCl_4$) *2 step* 1100 °C	348.4(2)	1156.9(7)	90	120	6.61	3.23	3.21	2.06
SSVG ($RhCl_3$) *oscill.* 1100 °C	348.0(1)	1155.3(5)	90	120	3.58	2.23	1.80	1.98
SSVG ($SeCl_4$) *oscill.* 1100 °C	348.4(1)	1157.0(4)	90	120	7.48	3.41	2.41	3.10

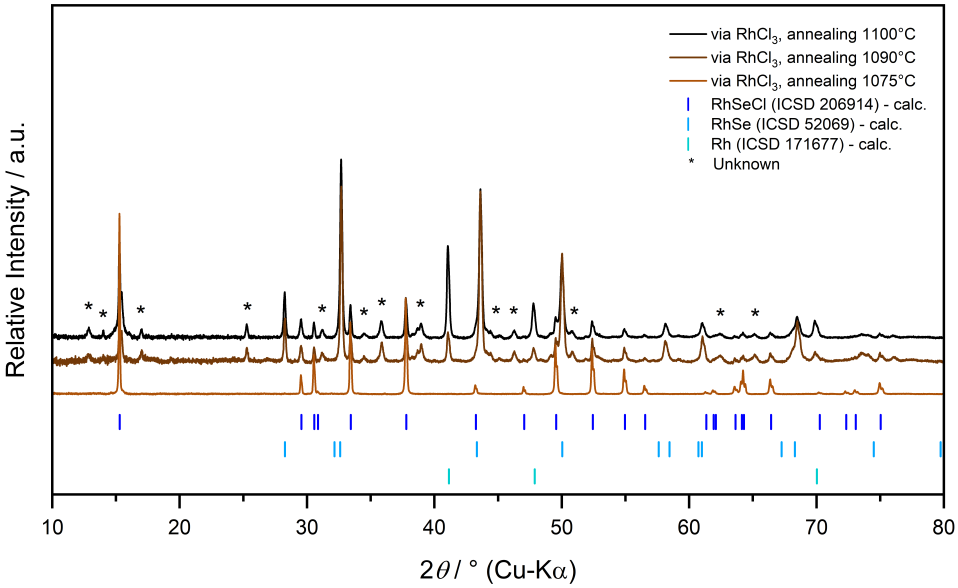

Figure 4: PXRD pattern of one-step SSVG syntheses in a tubular furnace with Rh, Se, and $RhCl_3$ as starting materials. It showcases that the two-step SSVG method is necessary: the black pattern represents the synthesis at 1100 °C, the dark brown-colored pattern at 1090 °C, and the light brown-coloured pattern at 1075 °C. The references from the ICSD are shown below with blue lines indicating the respective positions of the reflections. The asterisk indicates a currently unknown phase. The backgrounds of all experimental patterns have been evened out in the software Match! through subtraction of the capillary and starch reference background to ensure an easier comparison of experimental patterns.

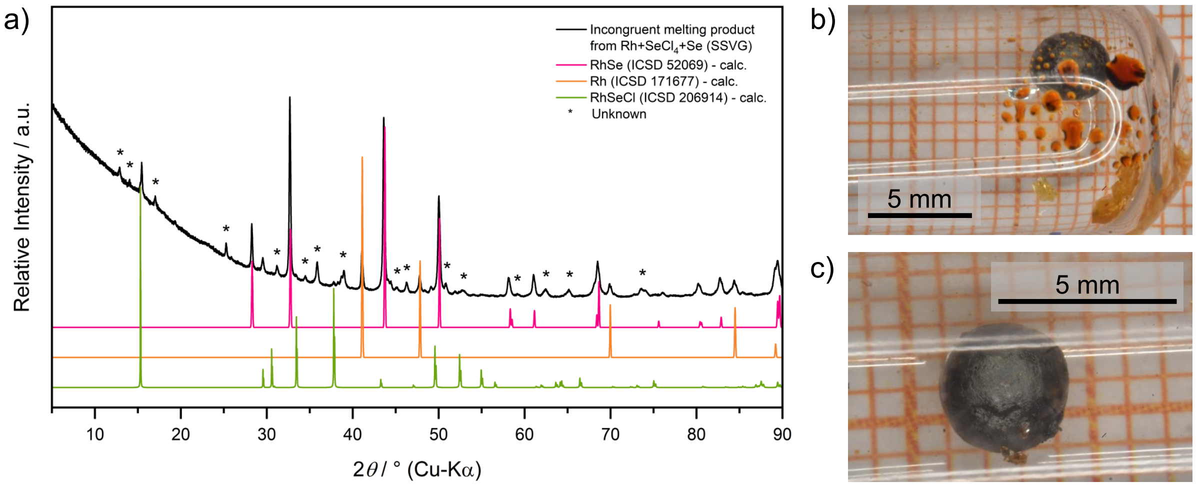

Figure 5: The incongruent melting of the RhSeCl if the ampoule is opened in between the two SSVG steps. The PXRD pattern in (a) showcases the products following the second SSVG step: the black pattern represents the measured data, the colored ones the comparative references from the ICSD (pink for RhSe, orange for Rh, and green for RhSeCl). The asterisks represent a currently unknown phase. Optical microscope images: (b) the resulting orange droplets of the Se- and Cl-based phase and the molten 'ball', (c) a closer view of the 'ball'.

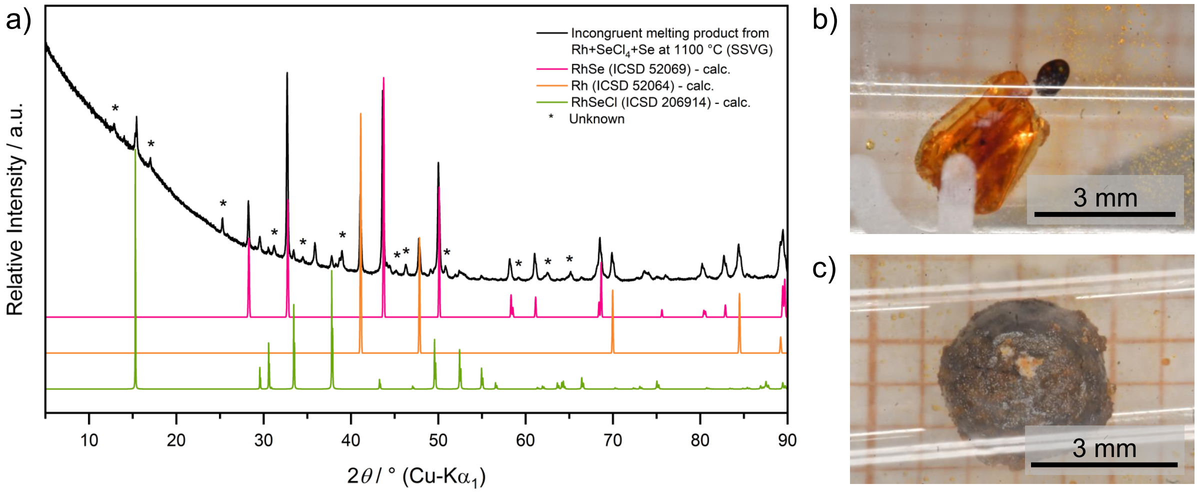

Figure 6: The incongruent melting of the RhSeCl if the ampoule is quenched at 1100°C in the second SSVG step. The PXRD pattern in (a) showcases the products following the second SSVG step: the black pattern represents the measured data, the colored ones the comparative references from the ICSD (pink for RhSe, orange for Rh, and green for RhSeCl). The asterisks represent a currently unknown phase. The optical microscope images in (b) and (c) show the view of the ampule before opening. (b) The resulting orange crystals of the Se- and Cl-based compound. (c) The 'ball' of the melt.

Single Crystal X-ray Diffraction

Table 2: Crystallographic data for single-crystal X-ray diffraction of RhSeCl

**Crystallographic data**				
Synthesis Method	CVT	SSVG	SSVG	SSVG oscill.
Precursor	$SeCl_4$	$RhCl_3$	$SeCl_4$	$SeCl_4$
Composition	— RhSeCl —			
Structure type	— CdI_2(hP6) —			
Crystal system	— Hexagonal —			
Space group	— $P6_3mc$ (No. 186) —			
$a = b$ / pm	349.12(3)	348.72(1)	348.45(2)	349.15(3)
c / pm	1158.41(11)	1153.81(5)	1156.00(10)	1158.29(12)
V / $\times 10^6$pm^3	122.28(2)	121.512(9)	121.554(17)	122.28(2)
Formula Units, Z	2	2	2	2
Calc. ρ (g cm^{-3})	5.903	5.940	5.938	5.902
**Data Collection**				
Temperature / K	200(1)	100(1)	250(1)	250(1)
Radiation	— Mo-Kα, $\lambda = 71.073$ pm —			
Crystal size / mm^3	$0.06 \times 0.08 \times 0.13$	$0.049 \times 0.057 \times 0.068$	$0.15 \times 0.18 \times 0.26$	$0.07 \times 0.14 \times 0.53$
Range of Collection / °	$3.518 \leq \theta \leq 37.399$	$3.532 \leq \theta \leq 37.457$	$3.525 \leq \theta \leq 37.728$	$3.518 \leq \theta \leq 37.396$
h range	$-5 \leq h \leq 5$	$-5 \leq h \leq 5$	$-5 \leq h \leq 6$	$-5 \leq h \leq 5$
k range	$-5 \leq k \leq 5$	$-5 \leq k \leq 5$	$-6 \leq k \leq 5$	$-5 \leq k \leq 5$
l range	$-19 \leq l \leq 19$	$-19 \leq l \leq 19$	$-19 \leq l \leq 19$	$-19 \leq l \leq 19$
**Data Reduction**				
Reflections	3627 / 299	3342 / 297	3397 / 298	3246 / 300
Absorption correction	— Numerical, Gaussian grid —			
μ / mm^{-1}	22.5	22.7	22.7	22.5
F(000)	192	192	192	192
Transmission	0.21 to 0.51	0.56 to 0.69	0.03 to 0.22	0.87 to 1.89
**Refinement Parameters**				
Data / Parameter / Restraint	299 / 10 / 0	297 / 11 / 0	298 / 11 / 0	300 / 10 / 0
R_{int} / %	0.0986	0.0483	0.1286	0.1214
R_σ / %	0.0340	0.0249	0.0491	0.0325
R_1 ($F_o > 4\sigma(F_o)$) / %	0.0473	0.0190	0.0423	0.0574
R_1 (all data) / %	0.0520	0.0218	0.0475	0.0603
wR_2 (all F_o) / %	0.1219	0.0407	0.1171	0.1618
$GooF$	1.200	1.151	1.285	1.318
Resid. Electron Density (e $\times 10^{-6}$ pm^{-3})	− 1.72 to 1.50	− 0.87 to 1.12	− 1.61 to 3.06	− 2.33 to 2.04

Table 3: Wyckhoff position, coordinates, coefficients U_{ij} (/ pm^2) of the tensors of the anisotropic displacement factor, and the equivalent displacement factor for the atoms in RhSeCl grown via the CVT method from $SeCl_4$ at 100(1) K. U_{eq} is defined as one third of the trace of the orthogonalized U_{ij} tensor.

Atom	W·P·	x	y	z	s·o·f·	U_{11}	U_{22}	U_{33}	U_{23}	U_{13}	U_{12}	U_{eq}
Rh	2b	2/3	1/3	0.49253(2)	0.16667	67.8(41)	67.8(41)	101(5)	0	0	33.9(21)	78.9(34)
Se	2b	1/3	2/3	0.37919(16)	0.16667	80.3(63)	80.3(63)	86.8(72)	0	0	40.2(32)	82.5(44)
Cl	2a	1	0	0.62133(41)	0.16667	87.7(97)	87.7(97)	117.6(13)	0	0	43.9(48)	97.7(67)

Table 4: Wyckhoff position, coordinates, coefficients U_{ij} (/ pm^2) of the tensors of the anisotropic displacement factor, and the equivalent displacement factor for the atoms in RhSeCl grown via the SSVG method from $RhCl_3$ at 100(1) K. U_{eq} is defined as one third of the trace of the orthogonalized U_{ij} tensor.

Atom	W·P·	x	y	z	s·o·f·	U_{11}	U_{22}	U_{33}	U_{23}	U_{13}	U_{12}	U_{eq}
Rh	2b	1/3	2/3	0.49239(3)	0.16667	26.4(22)	26.4(22)	47.5(29)	0	0	13.2(11)	33.4(16)
Se	2b	2/3	1/3	0.37903(9)	0.16667	28.5(32)	28.5(32)	55.1(44)	0	0	14.3(16)	37.4(22)
Cl	2a	0	1	0.62116(22)	0.16667	50.7(48)	50.7(48)	53.0(74)	0	0	25.3(24)	51.5(33)

Table 5: Wyckhoff position, coordinates, coefficients U_{ij} (/ pm^2) of the tensors of the anisotropic displacement factor, and the equivalent displacement factor for the atoms in RhSeCl grown via the SSVG method from $SeCl_4$ at 250(1) K. U_{eq} is defined as one third of the trace of the orthogonalized U_{ij} tensor.

Atom	W·P·	x	y	z	s·o·f·	U_{11}	U_{22}	U_{33}	U_{23}	U_{13}	U_{12}	U_{eq}
Rh	2b	2/3	1/3	0.50744(2)	0.16667	82.3(46)	82.3(46)	134.9(58)	0	0	41.2(23)	99(4)
Se	2b	1/3	2/3	0.62057(18)	0.16667	90.9(65)	90.9(65)	128(8)	0	0	45.4(33)	103.3(48)
Cl	2a	1	0	0.37861(45)	0.16667	118.3(97)	118.3(97)	149(14)	0	0	49.1(49)	128.5(71)

Table 6: Wyckhoff position, coordinates, coefficients U_{ij} (/ pm^2) of the tensors of the anisotropic displacement factor, and the equivalent displacement factor for the atoms in RhSeCl grown via the SSVG oscillating method from $SeCl_4$ at 250(1) K. U_{eq} is defined as one third of the trace of the orthogonalized U_{ij} tensor.

Atom	W·P·	x	y	z	s·o·f·	U_{11}	U_{22}	U_{33}	U_{23}	U_{13}	U_{12}	U_{eq}
Rh	2b	1/3	2/3	0.50684(3)	0.16667	75.3(55)	75.3(55)	191.9(72)	0	0	37.7(28)	114.2(46)
Se	2b	2/3	1/3	0.62006(23)	0.16667	92.5(84)	92.5(84)	171(10)	0	0	46.3(42)	119(6)
Cl	2a	0	1	0.37802(61)	0.16667	117(13)	117(13)	219(19)	0	0	58.7(68)	151.3(95)

EDS Analysis

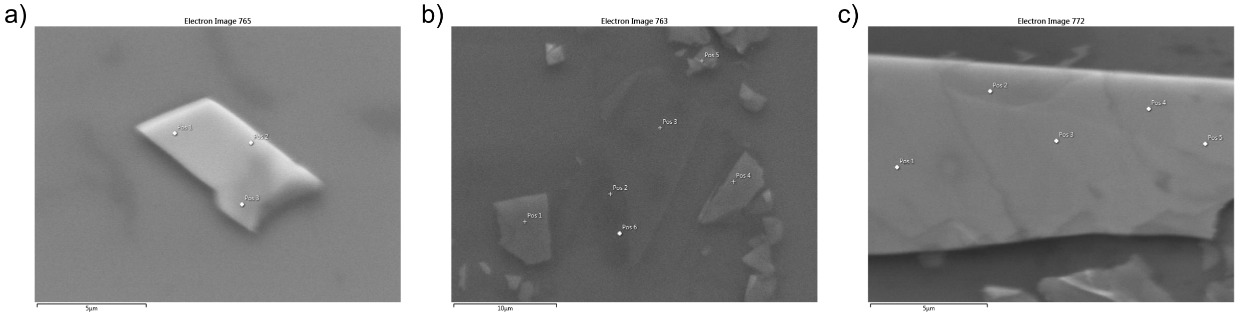

Figure 7: SEM images of: (a) bulk flakes of crystals grown via the SSVG method employing $RhCl_3$ as a reagent (see Table 7); (b) few-layer flakes exfoliated from crystals grown via the two-step SSVG method employing $RhCl_3$ as a reagent (see Table 8); and (c) few-layer flakes exfoliated from crystals grown via the two-step SSVG method employing $SeCl_4$ as a reagent (see Table 9).

Table 7: EDS data for bulk flakes of RhSeCl grown via two-step SSVG with $RhCl_3$.

**Spectrum**		**Atomic %**	
**Label**	**Rh**	**Se**	**Cl**
Pos 1	32.56	32.58	34.86
Pos 2	33.29	32.19	34.52
Pos 3	33.17	32.97	33.86
**Statistics**			
Maximum	33.29	32.97	34.86
Minimum	32.56	32.19	33.86
Average	33.01	32.58	34.41
Standard Deviation	0.39	0.39	0.51

Table 8: EDS data for few-layer flakes of RhSeCl grown via two-step SSVG with $RhCl_3$.

**Spectrum**		**Atomic %**	
**Label**	**Rh**	**Se**	**Cl**
Pos 1	23.62	2.52	73.87
Pos 2	14.23	26.22	59.55
Pos 3	24.21	11.34	64.44
Pos 4	22.94	2.20	74.86
Pos 5	23.56	4.46	71.97
Pos 6	30.97	20.19	48.84
**Statistics**			
Maximum	30.97	26.22	74.86
Minimum	14.23	2.20	48.84
Average	23.26	11.16	65.59
Standard Deviation	5.33	10.08	10.14

Table 9: EDS data for few-layer flakes of RhSeCl grown via two-step SSVG with $SeCl_4$.

**Spectrum**		**Atomic %**	
**Label**	**Rh**	**Se**	**Cl**
Pos 1	33.00	32.93	34.07
Pos 2	33.47	32.92	33.61
Pos 3	33.05	33.09	33.85
Pos 4	32.63	32.94	34.43
Pos 5	32.61	32.97	34.42
**Statistics**			
Maximum	33.47	33.09	34.43
Minimum	32.61	32.92	33.61
Average	32.95	32.97	34.08
Standard Deviation	0.35	0.07	0.36
